# Ken Donaldson: retirement of a young mind

**DOI:** 10.1186/1743-8977-10-8

**Published:** 2013-03-26

**Authors:** Paul Borm, Flemming R Cassee

**Affiliations:** 1 Centre of Expertise in Life Sciences, Zuyd University, Heerlen, The Netherlands; 2 Centre of Sustainability, Environment and health, National Institute of Public health and the Environment, Bilthoven, The Netherlands

## 

On 8 April 2013, Ken Donaldson of the MRC/University of Edinburgh Centre for Inflammation research and ELEGI Colt lab, is retiring from his current job. Professor Donaldson - Ken to most of those who know him - is one of the few particle toxicologists left who originally saw and investigated the three major problems in particle toxicology. These are coal-mine dust, quartz and asbestos [1,2].

He began his career as a medical laboratory technician at the University of Edinburgh in 1966 and took up a position as a scientist at the Institute of Occupational Medicine (IOM) in Edinburgh, UK, in 1981. He then quickly realized his unique capacity to translate environmental and occupational problems into simple research questions and – even more importantly - easy operational experiments to answer the questions put forward. At the IOM, Ken worked on major health-effects associated with exposure to coal, quartz and asbestos and introduced the first mechanistic work to complement the vast epidemiological and exposure database. Inspired by the work and vision of John Davies, Chris Wagner and later Anthony Seaton, he gradually took his position as world-leading expert in particle and fibre toxicology. The success of the research in coal mine and quartz was associated with better conditions in the coal mines. The closing of most European coal mines in the 1880 s due to economic issues made further research in this area redundant. In addition, the IOM had to refocus and reorganize finding new targets and a new mission.

During this transition period in particle toxicology, Ken Donaldson moved to (what is now called) Edinburgh Napier University in 1992 to become a reader in pathobiology, in reality only 150 yards away from the IOM-nest. In the meantime, a new particle problem was emerging, ambient particulate matter, rising from the famous six cities study from Dockery and co-workers [3]. The old IOM team (Donaldson and Seaton) developed some new clinical insights. A ground-breaking paper in the Lancet [4] was born, connecting the rapidly expanding field of research on ambient particles (PM) to clinical pathways in atherosclerosis as well as cardiac disease, using inflammation as a common pathway. Like all toxicologists, it took a change of mind to realize that small particles in microgram dose could cause death in vulnerable subjects, as compared to the grams of coal mine dust that diseased and killed many underground miners.

In 1995, Ken was appointed professor in pathobiology at Napier and in 2002 he moved to the University of Edinburgh to join his friend William (Bill) MacNee. Together with Günther Oberdorster, he was one of the first to realize that the very small particles in air (‘ultrafines’) may possess specific features that discriminate these from the larger yet respirable particles [5].

Although sceptical until the very end about the real significance of nanomaterials in terms of their impact on health, Ken has contributed significantly to the development of toxicology of nanomaterials. From his know-how and experience with asbestos and man-made fibres, he was the first to extrapolate the Stanton theory to carbon nanotubes and gave it an extra dimension (i.e. that of length, diameter, rigidity and biopersistence) all crucial parameters to predict the effects of these fibres, and defining the term HARN, high-aspect ratio nanoparticles. In a paper with Craig Poland that stirred the nano-world, Ken was able to show that stiff, rigid carbon nanotubes simply follow the toxicity paradigm as previously elaborated by his idols Chris Wagner and John Davies [6]. Although the existing paradigm was confirmed by his group in a set of elegant experiments, the nano-world was shocked to see that the hallmark of nanotechnology (carbon nanotubes) could behave like asbestos if certain conditions were met. And once again, Ken showed his admirable knowledge and didactic skill in the many research papers and reviews that he has published with his colleagues.

Paul commented: “The first time I really learned to know Ken was during the Particle Toxicology meeting in Lake Placid (USA) in 1996, organized by Kevin Driscoll and Günter Oberdörster. We were asked to organize the next meeting and decided to have this in Maastricht in 1999. During the preparation, Ken showed on numerous occasions that he is able to bring the profession forward by discussions, new hypotheses and his straightforward thinking. After many mutual visits in our labs and homes in Dusseldorf and Edinburgh, we decided to start a new particle journal in 2004. In September 2004, we launched *Particle and Fibre Toxicology* with BioMed Central as publisher took and our first steps in Open Access publishing [7].” More or less reflecting Ken’s career, the journal also intended to bring together all aspects of toxicological effects of particles and fibres, and extend the know-how in this area by exploring common mechanisms and denominators. With this journal and its current impact factor, *Particle and Fibre Toxicology* has rightfully claimed its position in science, and offers a platform for the diverse community that is involved in the global research enterprise in this field.

In 2007 Paul Borm and Ken decided “to do” a book on particle toxicology. On that occasion, while sitting in his office in Edinburgh, Ken made a statement “that this was among one of the things on his list he wanted to do” [8].

Donaldson sets an example to us all in his career path, unique findings and persistence and especially in the way he achieved those things in all modesty, keeping things simple and transparent. His unique contribution to particle and fibre toxicology has been to bridge the different particle era’s and each time, to save and increase the know-how in the field. His comments during meetings, workshops and in manuscript reviews rarely dealt with details but always had the mission to improve our profession and our knowledge. He remained very modest, yet seemed to always think a step ahead of the larger research community. We like to say and remember that he is the 15th most-cited author in air pollution research and the only toxicologist in this list of Thomson Essential Science Indicators.

Now Ken Donaldson is into music, which is nothing new to those that had the honour to stay at his house and enjoy his hospitality. The room full of guitars is now more frequently used for the ultimate Beatle-remix. No doubt we will be hearing from him in the future, in the typical Kenman-style. We invite you to visit his website: willothewispstudio.com.

On behalf of all readers, editors and back-office staff of *Particle and Fibre Toxicology*, we would like to thank Ken for putting his inspiration into this ever challenging journal!
